# Network Selection: A Method for Ranked Lists Selection

**DOI:** 10.1371/journal.pone.0043678

**Published:** 2012-08-24

**Authors:** Luisa Cutillo, Annamaria Carissimo, Silvia Figini

**Affiliations:** 1 Department of Statistics and Mathematics for the Economic Research, University of Naples “Parthenope”, Naples, Italy; 2 Bioinformatics Core, Telethon Institute of Genetics and Medicine, Naples, Italy; 3 University of Pavia, Pavia, Italy; Aalto University, Finland

## Abstract

We consider the problem of finding the set of rankings that best represents a given group of orderings on the same collection of elements (preference lists). This problem arises from social choice and voting theory, in which each voter gives a preference on a set of alternatives, and a system outputs a single preference order based on the observed voters’ preferences. In this paper, we observe that, if the given set of preference lists is not homogeneous, a unique true underling ranking might not exist. Moreover only the lists that share the highest amount of information should be aggregated, and thus multiple rankings might provide a more feasible solution to the problem. In this light, we propose *Network Selection*, an algorithm that, given a heterogeneous group of rankings, first discovers the different communities of homogeneous rankings and then combines only the rank orderings belonging to the same community into a single final ordering. Our novel approach is inspired by graph theory; indeed our set of lists can be loosely read as the nodes of a network. As a consequence, only the lists populating the same community in the network would then be aggregated. In order to highlight the strength of our proposal, we show an application both on simulated and on two real datasets, namely a financial and a biological dataset. Experimental results on simulated data show that *Network Selection* can significantly outperform existing related methods. The other way around, the empirical evidence achieved on real financial data reveals that *Network Selection* is also able to select the most relevant variables in data mining predictive models, providing a clear superiority in terms of predictive power of the models built. Furthermore, we show the potentiality of our proposal in the bioinformatics field, providing an application to a biological microarray dataset.

## Introduction

In recent years, rank aggregation methods have emerged as an important approach able to combine the ranking information from different statistical units. In diverse interest areas, the rank aggregation process is usually devoted to the merging of different preference lists on the same set of units. Relevant applications are collected in marketing and advertisement research, applied psychology, internet search engines and more recently in omics scale biological studies. In the literature, this problem was first addressed by Arrow [1], Kemeny [2] and later, in terms of application to the World Wide Web data, by [3].

On the basis of our experience rank aggregation techniques are shown to be very informative also in the field of economic applications, especially in risk analysis and risk integration. In particular, given a set of statistical units (i.e. a set of enterprises) potentially at risk of failure, it would be highly interesting to order them using a collection of variables available. In this perspective, we think that rank aggregation methods lend naturally to the field of economic applications and thus we also show an application of our novel methodology to a real financial data set.

Despite its clear and intuitive target, effective rank aggregation becomes difficult in real-world situations in which the set of collected rankings can be noisy, incomplete, or even disjoint. The biggest challenges of the aggregating process remain today the choice of an appropriate measure of dissimilarity between lists, and a reasonable top 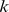 length for a particular list ([4], [5]). The classical rank aggregation techniques aim at merging different preference lists into a single final ordering on the same set of units. Unfortunately these procedures fail when the observed set of preference lists is heterogeneous. In order to overcome this weakness, we propose a methodological approach that directly take into account that a unique true underling ranking might not exist. Moreover we point out that only the lists that share the highest amount of information should be aggregated and only consensus sets of lists should be considered for the aggregation process. For sake of brevity, we introduce the acronym 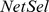 to refer to our *Network Selection* method.


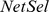
 is a heuristic rank aggregation method inspired by the graph theory. The rationale of this choice relies on the observation that, after a preprocessing step, we can loosely read our set of lists as a network. For an extensive review of the network theory we defer to [6]. The preprocessing step that we will describe later on, basically consists in choosing an appropriate measure of dissimilarity between lists and in performing an hypergeometric hypothesis test on each computed distance. This step leads to compute the adjacency matrix of the network whose nodes are the lists. The constructed network would then be partitioned via a standard communities extraction method [7]. Only the set of lists populating the same community in the network would then be aggregated. Communities, or clusters, can be considered as different compartments of a graph playing a similar role. Detecting communities is a very important interdisciplinary problem. A full exposition of this topic and the state of the art of the most method developed by scientists working on it can be found in [8].

Before describing our proposal, we introduce the general framework of the rank aggregation (RA) problem for a discrete set of statistical units. RA methods can be broadly classified as distributional based, stochastic optimization and heuristic algorithms [9]. The first category is populated by Thurstone’s method and its extensions. These methods reveal to be appropriate for aggregating many short ranked lists. Optimization algorithms are based on an optimization criteria and are usually dependent on the distance measure. In fact, given a distance measure, they aim to find the aggregate list as the candidate list that minimizes its distance from all the input lists. An instance of this category is the Kemeny optimal aggregation which optimizes the average Kendall’s distances [3]. Unfortunately it is well known that computing the Kemeny optimal aggregate is NP-hard even when the number of ranked lists to be aggregated is small; this is due to the combinatorial nature of the problem. These difficulties can be circumvented by stochastic search algorithms as described in [10].

A novel alternative to direct optimization is given by the heuristic algorithms that are capable of providing approximate solutions to the RA problem without optimizing any criterion. Effective applicative results of this heuristic category are shown in [3] and [4]. From a different point of view we can classify the RA methods according to the average length of the set of lists under study. The problem of aggregating many short lists is addressed by the distributional based and stochastic optimization algorithm, while the problem of aggregating a few long lists is tackled mainly by heuristic algorithms. The main limitation of all the 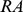 algorithms mentioned so far is the unfairness of the result for heterogeneous set of lists as in this scenario the aggregate list might be random. In particular it is reasonable to expect that many long lists represent a non homogeneous set of preferences. In the present paper we propose an innovative heuristic strategy that is particularly suited for the problem of aggregating a heterogeneous set of long lists.

## Methods

### Preliminaries

In the followings we introduce some necessary concepts and notations. Let 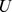 be a set of objects and consider a subset 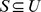 whose cardinality is 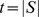. A ranking function on 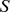 is a permutation 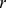 on the set 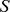. For each object 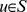, 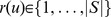 shows the ranking of item 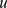. Of course a preference list on 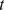 objects can be considered as a point in a 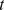
- dimensional space 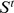 whose 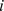-th component 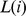 is the element of 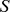 ranked at position 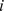. More precisely we say that 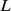 is a ranked list of the elements of 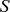 with ranking function 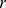, if the following relations holds:
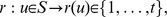


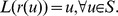



We will use the notation 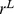 to refer to 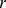, in order to explicit the linkage between the ordered list 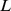 and it’s ranking function. Note that the best ranking is 1, rankings are always positive, and a higher rank corresponds to lower preference in the list. As an example consider the simple case in which a voter of 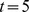 candidates 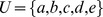 expresses the preference list 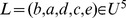. As a consequence the associated ranking function is such that: 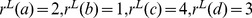 and 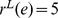.

**Table 1:** Scenario 1A average within distance and relative standard deviation for communities simulated with 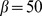 and 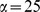.

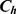	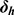	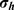
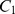	0.210159967	0.02889782
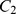	0.209791857	0.02889778
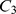	0.210035548	0.02895248
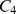	0.209975231	0.02892337

**Table 2:** Scenario 1A average between distance and relative standard deviation for communities simulated with 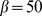 and 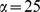.

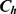	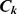	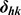	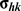
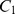	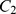	0.64152464	0.028919
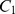	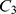	0.64156696	0.028832
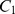	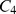	0.64155527	0.028768
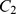	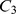	0.64174671	0.028965
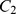	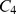	0.64177845	0.028846
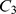	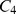	0.64156148	0.028891

A *full list* is a list that expresses a ranking for every item 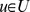, that is 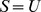. In this case its ranking function is a *complete ranking* on U. A *partial list* is a list that expresses rankings only for a proper subset of items 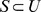. A partial list will be also referred to as a *Top-k* when 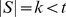.

Note that in this case we assume that all other items 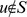 are supposed to be ranked below every item in 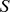 according to a customized ranking value. With a slight abuse of notation in the following by 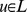 [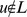] we mean 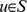 [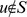]. Moreover we will often use 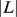 to mean the cardinality 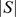 of the set of elements 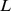 it is related. Given a set of complete or incomplete lists, we need to provide an approximate solution to their RA problem. In order to clarify the overall procedure described in the next subsection, we will briefly recall Borda-inspired methods and optimization methods.

**Table 3:** Scenario 2 average within distance and relative standard deviation for communities simulated with 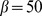 and 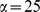.

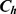	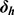	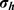
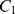	0.209805957	0.028877625
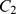	0.209939113	0.028840451
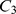	0.210040162	0.028895175
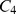	0.210083126	0.028848004

**Table 4:** Scenario 2 average between distance and relative standard deviation for communities simulated with 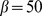 and 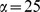.

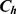	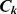	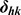	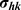
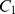	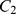	0.641569346	0.028915927
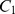	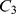	0.641501867	0.028864567
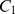	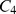	0.641801049	0.028848633
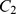	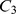	0.641560463	0.028739462
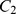	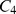	0.641760131	0.028849104
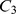	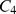	0.641406958	0.028983586

Borda-inspired algorithms are a family of intuitive and easy to understand RA methods that basically reproduce a voting strategy. Jean-Charles de Borda in 1781, originally proposed to aggregate ranks by sorting the ranks arithmetic average for full ranked lists [11]. Many other variations of the Borda method have been proposed and used, and are applicable to top-k lists.

Suppose we have 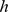 ordered complete lists 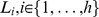 on 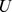, the Borda score associated with a generic element 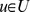 for the list 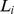 is 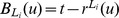 apart from an optional scaling factor. Borda’s score may in fact take into account other additional information than the rankings when available. Each element 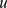 is then assigned an aggregate score that summarizes all the Borda’s scores from the 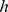 lists. This cumulative score is returned by an aggregating function 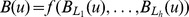 that specifies the law of aggregation of the 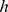 available scores for 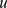. The aggregated ranked list is then obtained by sorting all the aggregated Borda’s scores in ascending order. In the original method proposed by Borda in 1781 the aggregation function was the arithmetic mean of all the Borda’s scores. This is a special case of the most general *p-norm*, when 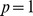:
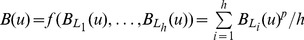
(1)


As example consider the case of 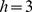 voters of 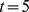 candidates 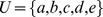. Suppose that the three voters produces the following full preference lists:
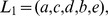


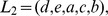


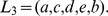



The corresponding Borda’s scores associated to the 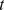-tuple 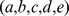 are respectively:
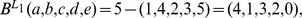


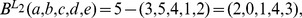


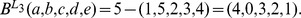



According to (1) the aggregate 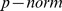 scores associated to the 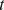-tuple 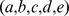, with 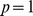, and the corresponding aggregate list 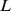 are:
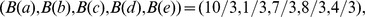


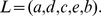



This is just a toy example to get familiar with the concept of lists and aggregate lists, thus we are not discussing about the goodness of the this aggregation. The extension of this method to the Top-k case is straightforward [11].

**Table 5:** Predictive models on the whole data set.

Model	decile	LIFT	CCR
Tree	1	2.77	27.71
Tree	2	2.54	53.07
Tree	3	1.56	68.70
Tree	4	0.57	74.36
Tree	5	0.46	78.93
Tree	6	0.46	83.49
Tree	7	0.46	88.05
Tree	8	0.46	92.62
Tree	9	0.46	97.18
Tree	10	0.28	100.00
Log Reg	1	0.09	0.86
Log Reg	2	0.69	7.76
Log Reg	3	0.34	11.21
Log Reg	4	0.60	17.24
Log Reg	5	0.86	25.86
Log Reg	6	1.38	39.66
Log Reg	7	2.07	60.34
Log Reg	8	0.95	69.83
Log Reg	9	1.55	85.34
Log Reg	10	1.47	100.00

On the other hand, optimization methods is a family of algorithms that address the RA problem in terms of an optimization rule. The most common optimization strategies are based on a measure of disagreement between the input top-k lists and the unknown aggregate rankings. One formulation that follows the generalized Kemeny criterion is the minimization of the weighted sum of distances between the aggregate rankings and the input lists. Thus, whether a particular aggregate list is better than another one, depends on the distance measure chosen. The most common distance measures between lists are the footrole and the kendall.

Given two lists 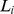 and 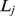 on the same set of elements 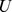, the footrule distance 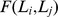 between them is defined to be 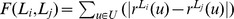. This distance expresses a sort of total absolute deviation of the two lists on single elements but does not take into account the relative orderings of each couple of elements. The Kendall tau distance 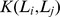 between 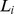 and 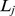 is the number of couples of elements 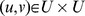, such that either 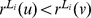 but 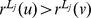, or 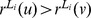 but 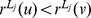.

It is easy to see that 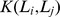 measures the number of pairwise disagreements between the two lists. Observe that the number of disagreements (MISMATCHES) and agreements (MATCHES) between two complete lists of same length 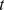 is such that:
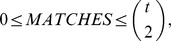


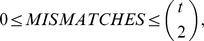


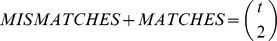



A Kendall optimal aggregation of the given set of lists is any aggregate list 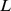 that minimizes 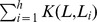; similarly, a footrule optimal aggregation is any list L that minimizes 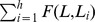. As previously noticed, computing a Kendall optimal aggregation is NPhard, while computing a footrule optimal aggregation can be done in polynomial time via minimum cost perfect matching ([3]).

Nevertheless, in the majority of the cases, it is of higher interest to provide an aggregate list that accounts for the most frequent pairwise agreements in the set of input lists.

**Figure 1:**
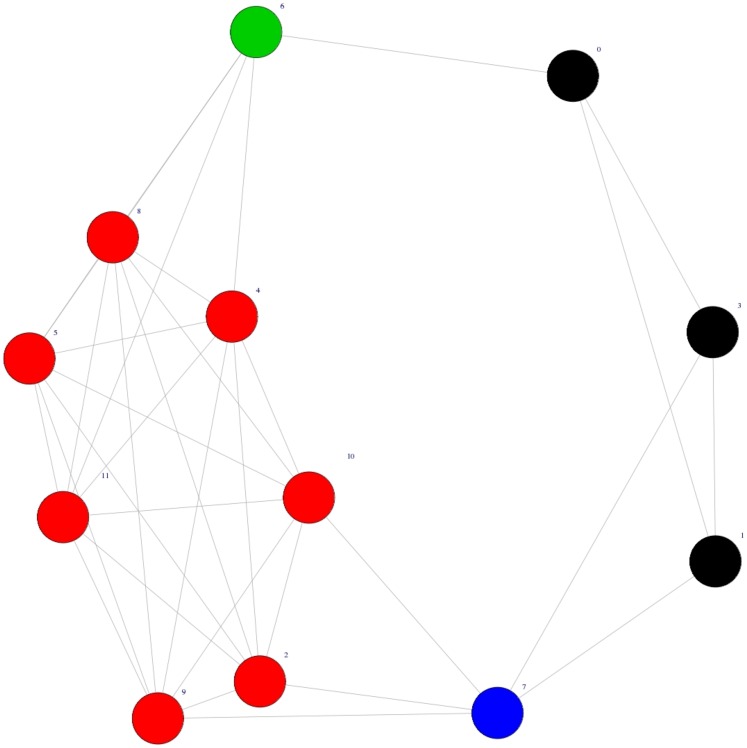
*NetSe* *l* network on the real dataset. The relative exctracted communities are C1(black dots), C2 (red dots) and the two outliers variables C4 (blue dot) and C3 (green dot).

**Table 6:** *NetSel* communities extraction result on the proposed set of financial ratios.

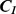	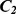	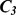	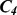
Supplier target days	Liquidity ratio	Cost income ratio	Trade payable ratio
Outside capital strucure	Cash ratio		
Capital tied up	Equity ratio		
	Cash flow to effective debt		
	Liabilities ratio		
	Result ratio		

**Table 7:** *NetSel* extracted communities within distance and relative standard deviation.

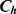	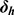	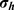
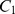	0.341511596	0.006955172
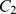	0.33071787	0.11191681

**Table 8:** Average distance between the *NetSel* extracted communities and relative standard deviation.

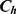	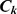	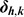	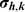
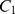	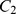	0.605087116	0.04946876
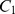	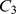	0.500418	0.105093445
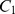	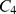	0.394367666	0.141355664
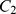	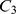	0.500584162	0.060114893
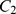	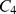	0.560789719	0.093720458
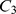	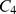	0.662595709	NA

### Our Proposal

In the following we describe the main aspects of our contribution that result in a novel algorithm able to tackle a non homogeneous large set of long lists. The main target of 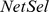 is to find the subgroups of homogeneous rankings. This is motivated by the observation that in real world cases, as in politics, there exists few general trends that govern the preference expressions. As a consequence only preferences in high agreement should contribute to the formation of a single list that summarizes the common unknown trend. 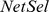 overall procedure can be broadly summarized in four steps.

**Table 9:** Predictive models on 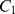.

Model	decile	LIFT	CCR
Tree	1	4.32	43.21
Tree	2	1.25	55.70
Tree	3	0.84	64.06
Tree	4	0.84	72.42
Tree	5	0.84	80.78
Tree	6	0.81	88.91
Tree	7	0.29	91.85
Tree	8	0.29	94.79
Tree	9	0.29	97.73
Tree	10	0.23	100.00
Log Reg	1	3.00	30.00
Log Reg	2	2.17	51.72
Log Reg	3	0.95	61.21
Log Reg	4	0.69	68.10
Log Reg	5	0.86	76.72
Log Reg	6	0.52	81.90
Log Reg	7	0.86	90.52
Log Reg	8	0.34	93.97
Log Reg	9	0.34	97.41
Log Reg	10	0.26	100.00

**Table 10:** Predictive models on 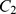.

Model	decile	LIFT	CCR
Tree	1	3.17	31.70
Tree	2	2.08	52.47
Tree	3	1.94	71.85
Tree	4	0.94	81.29
Tree	5	0.72	88.51
Tree	6	0.37	92.23
Tree	7	0.37	95.94
Tree	8	0.37	99.66
Tree	9	0.03	100.00
Tree	10	0.00	100.00
Reg	1	3.10	31.03
Reg	2	1.98	50.86
Reg	3	1.72	68.10
Reg	4	1.21	80.17
Reg	5	0.60	86.21
Reg	6	0.60	92.24
Reg	7	0.34	95.69
Reg	8	0.34	99.14
Reg	9	0.09	100.00
Reg	10	0.00	100.00

The **first step** considers the allocation of a distance matrix between the lists. In order to aggregate a given set of lists, it is required to define a degree of similarity between them. To reach this objective, we have to introduce a similarity-dissimilarity measure between couples of lists. If we interpret each list as a point in a multidimensional space, this measure reveals to be a distance. Despite existence of several standard methods to define a distance measure between two lists, we choose the Kendall’s tau metric. This is due, as previously noticed, to its capability of accounting for the most frequent pairwise agreements in the set of input lists. Indeed it is reasonable to think that in a homogeneous set of lists the majority of elements share the same relative ordering and not the same exact ordering. Suppose we have 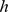 ordered lists 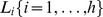 whose lengths, 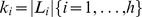, are not necessarily the same. We create a distance matrix according to a modified version of the Kendall’s tau distance [5]:
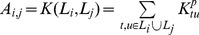
(2)where 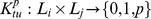 is a piece-wise function of the relative orderings (1) defined as follows:



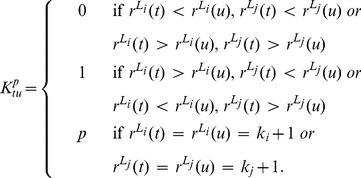
(3)Our choice of 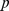 relies on the 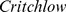 criterion [12].

**Figure 2:**
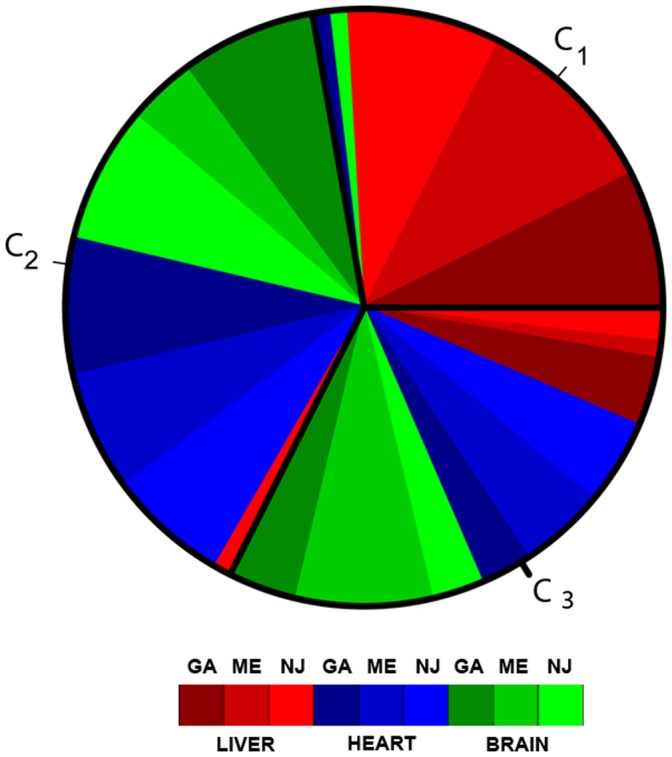
Pie plot of the percentage of tissue samples assigned by *NetSel* to each community detailed in **Table 11**. The green dye indicates the brain, the red dye indicates the liver and the blue dye indicates the heart. Each color has three intensities: light for the New Jersey (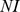), medium for the Maine (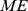) and dark for the Georgia (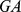).

**Table 11:** Percentage of tissue samples assigned by 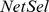 to each community.

tissue/community	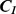	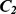	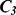
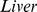	0.78	0.03	0.19
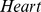	0.03	0.61	0.36
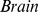	0.03	0.555	0.415

The **second step** consists of translating the distance matrix into the adjacency matrix of an undirected graph. Let 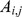 be the generic element of the distance matrix obtained so far. 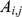 shows us how dissimilar list 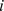 and list 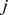 are, but we want to be more strict on the concept of dissimilarity. In this perspective we reduce the distance matrix 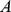 to a 0–1 adjacency matrix 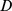 of an undirected graph where each vertex is a list. This is achieved via an hypothesis test on the match value of each couple of lists as explained in the following. Given a couple of lists of length 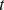, we test the null hypothesis 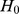 that the two lists are dissimilar versus the alternative 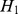 that the two lists are similar. In order to perform the test, we have to specify the distribution of the number of matches under the null hypothesis. We observe that it is reasonable to consider two lists dissimilar when, given any couple of elements of 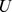, they have the same probability to be a match or a mismatch between the two lists. Moreover, when counting the number of matches between two lists of length 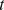, we are evaluating 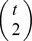 couples of elements. Under this perspective we can consider the number of matches a the result of a statistical experiment that has the following properties:

- A sample of size 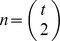 is randomly selected without replacement from a population of 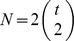 items.
- In the population, 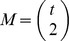 items can be classified as successes (matches), and 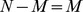 items can be classified as failures (mismatches).

Note that the condition 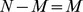 is due to the assumption of equiprobability of matches and mismatches. It is easy to conclude that, under 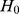, the measured number of matches is the realization of the hypergeometric random variable of the distribution:
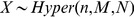
(4)with parameters 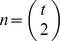, 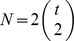 and 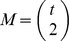.

In particular let 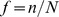 be the sampling fraction and let 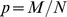 denote the proportion of matches in the population. Normal approximations to Hypergeometric distribution are classical in the standard cases where 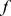 and 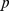 are bounded away from 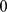 and 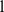 [13]. Thus under 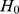 we approximate the hypergeometric distribution with the Normal distribution with mean 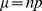 and variance 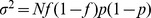.

**Figure 3:**
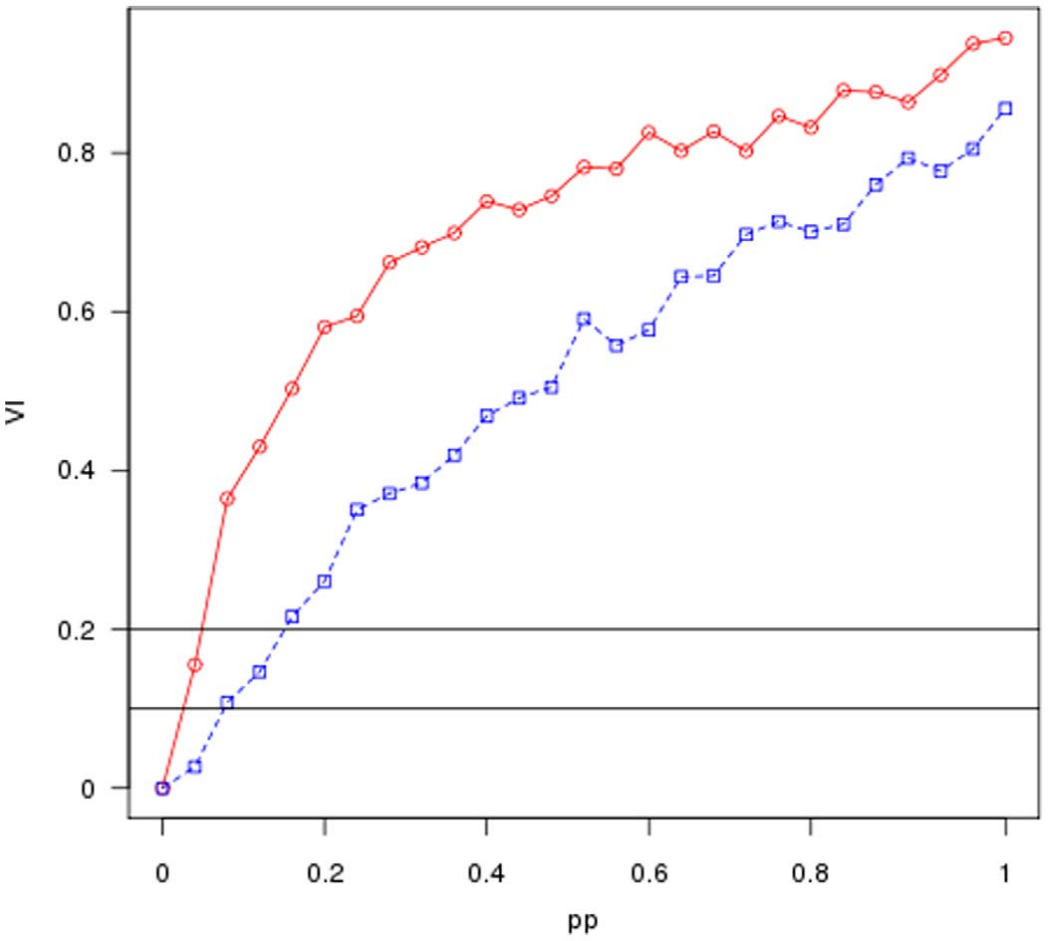
Average results of the application of our stability analysis to the real finacial data example. The average value of the normalized variation of information is plotted as a function of the amount of perturbation 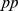. The black points in the figures show the variation of information for the unperturbed financial network while the red points show the results for the correspondent random graph (null model).

**Figure 4:**
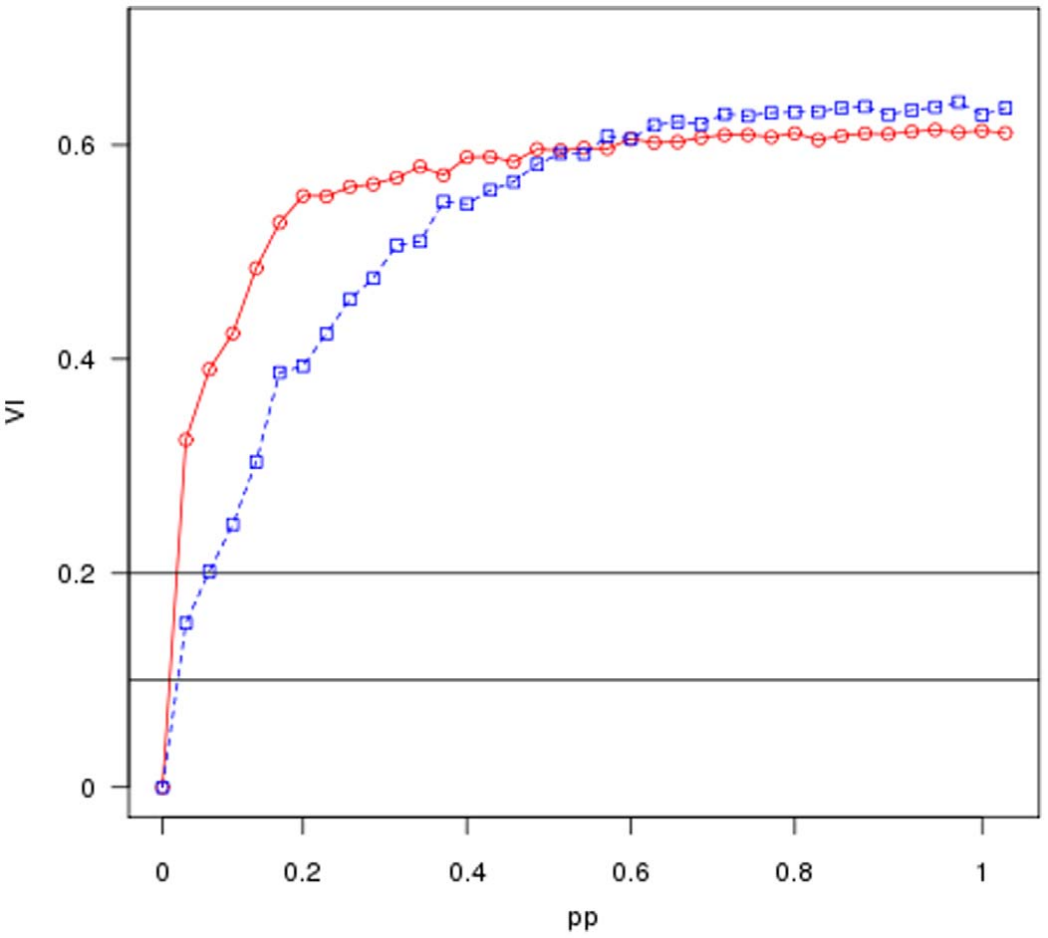
Average results of the application of our stability analysis to the real Biological data example. The average value of the normalized variation of information is plotted as a function of the amount of perturbation 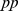. The black points in the figures show the variation of information for the unperturbed biological network while the red points show the results for the correspondent random graph (null model).

For each 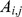 the corresponding 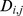 would be set either to one, if the null hypothesis is rejected, or to zero otherwise. In the rejection procedure the false discovery rate is controlled at level 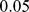 via the classical Benjamini-Hockberg procedure [14]. In practice the condition 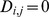 suggests that lists 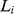 and 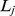 should not be aggregated together because they express discordant preferences and thus forcing them in the aggregation process would add noise to the final aggregate list. The other way around the eventuality 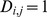 suggests that lists 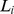 and 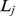 are in high agreement and thus might be close to the same underling true ranking. This step crucially transforms our set of lists into an undirected graph. In the case of a heterogeneous set of lists the adjacency matrix of this graph would be very sparse. The sparsity is a desirable property, that would allow to easily find the outliers. Indeed an outlier list would be translated into an isolated node. Moreover in a sparse network it is more intuitive to find the groups of most similar lists as the most densely connected subsets of nodes, as described in the next step.

The **third step** is devoted to the extraction of communities of similar lists from the network constructed in the second step. The adjacency matrix built so far would in fact be used to individuate the set of similar lists and, as said, to eventually isolate outliers. This is carried out through a community extraction algorithm as we assume our list network consists of modules which are densely connected themselves but sparsely connected to other modules. In this light we performed the community structure detection via 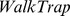 a standard algorithm based on random walks [7]. This third step outputs a clustering of our set of lists. We recall that a clustering 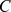 is a partition of a given set of elements (lists), into disjoint subsets 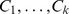 called clusters. In our case the extracted communities form indeed a clustering.

As pointed out in the 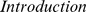 section, scientist devote huge effort in developing methods for community detection [8] hence the 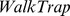 algorithm employed in the third step of 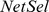 has been chosen among a variety of available community detection methods. In the subsection 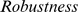 we will show the stability of 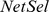 with respect to the specific clustering method chosen. Actually we could also find the groups of similar lists clustering them according to the distance matrix 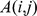. As we will see in the results section, this would lead to a similar result in terms of number of communities but would not isolate the outliers. More rigorous statistical models devoted to the clustering of infinite rankings have been developed [15]. Despite its innovative approach and excellent results, the model proposed in [15] is suited for 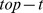 orderings, with 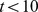. Our overall empirical procedure is suited for complete or incomplete rankings with an arbitrary length 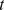, even 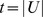.

The goal of the last and **fourth step** is to provide a consensus aggregate list for each of the extracted communities according to the third step. The aggregation is performed via a standard literature aggregation method for partial lists. We choose the Borda’s method (voting strategy) that, as said in the previous sections, has a very low computational cost and reveals to be efficient on a homogeneous set of lists. This last step is not crucial and is provided just for completeness. This is because our paper focuses on isolating homogeneous groups of lists and not on aggregating a homogeneous group of lists. Of course a comparison of aggregating methods for homogeneous rankings is out of the scope of the present work.

Our strategy enables to isolate outliers in a set of heterogeneous lists and tells which are the community of lists sharing the same information. For each community this information is provided by the list resulted from the aggregation step summarizing and representing the overall community. 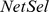 also provides a set of indicators that would suggest which communities are more representative of the underling observed units. Suppose we detected 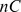 communities 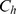, and assume that each community has size 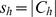, with 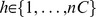. Our indicators are defined as follows:
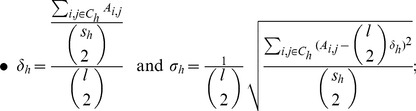




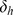
 gives the average percentage of mismatches within the same community and the 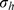 is its standard deviation.
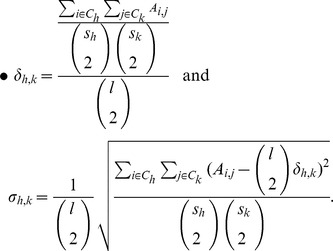



On the other hand 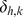 provides the average percentage of mismatches between each couple of identified communities 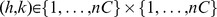 and 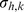 expresses its standard deviation.

Notice that, the most representative communities will be the ones with the smallest 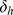 and the smallest 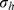. Moreover, in the best scenario, the most representative communities (say 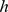 and 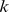), would also reveal to be well separated in the sense that 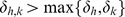 and 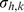 is small.

## Results

### Simulations

In this section we show the performance of 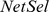 on simulated data sets. In order to control the ability of the method to recover the truth, we generated 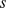 underling true rankings (*generating lists*), that is a generating list for each community. We allowed the dissimilarity between them to be 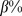 in terms of mismatches, with 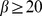. Each community was then populated by lists with 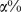 of disagreement from the relative generating one, with 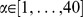.

The desired distances were reached composing two possible source of mismatches, *inversion* and *block exchange*, as defined in the following.

**Definition 1** Given a ranking function 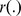 on a list 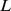, we define inversion 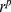 the ranking of 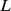 obtained by the permutation that expresses the reverse ordering of 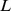 with respect to 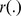:
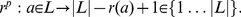
(5)


Observe that the ranked list resulting from the application of the inverse ranking 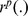 on the lists 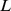 reaches the maximum number of mismatches with 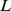, that is 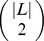.

**Definition 2** Given a ranking function 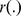 on a list 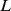, suppose it is possible to divide integrally the ranked list 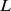 in 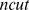 consecutive sublists (or blocks) 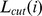, with 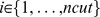. Assume that each block consists of 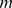 consecutive elements. We define block exchange of jump 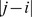 the exchange of the rankings of all the elements of block 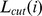 with the rankings of the corresponding elements of block 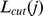, for 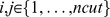. That is we define the new ranking 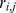 as follows:
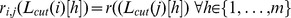


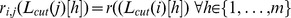


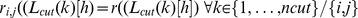



When 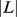 is not integrally divisible by 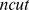, this definition can be trivially extended if the residual elements are included in the blocks external to 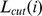 and 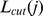. Note that the application of a *block exchange* of jump 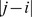 on a list 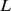, produces a number of mismatches with respect to the original list equal to 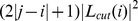.

In order to check the performance of the proposed algorithm, we need to establish a degree of similarity between the partition delivered and the true partition that we wish to recover. An accurate description of similarity measures for graph partitions can be found in [8]. The results from our simulations are summarized in terms of Variation of Information (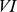), a novel criterion for comparing clusterings introduced in [16]. To understand this criterion, we need to introduce some basic concepts. Suppose that 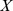 and 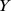 are the random variables describing two generic partitions on the same graph 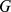. Let 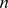 be the number of graph vertices, 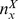 and 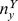 be respectively the number of vertex in clusters 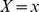 and 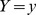 and let 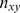 be the number of vertex shared by clusters 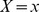 and 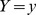. Assume that the random variables 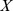 and 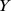 have joint distribution 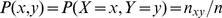, which implies that 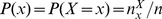 and 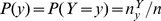. The 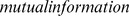 [17] between 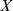 and 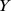 is defined as:
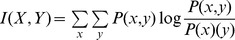
(6)


This measure is defined for two generic random variables and tell how much we learn about 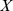 if we know 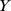 and viceversa. Actually 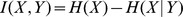, where 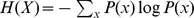 is the Shannon entropy of 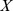 and 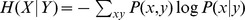 is the conditional entropy of 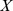 given 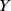 [17]. Melia [16] introduced the Variation of Information between the two clusterings as:
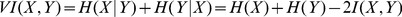
(7)


It can be shown that the 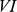 has the property of a distance and hence it defines a metric in the space of partitions. Moreover if two partitions differ only in a small portion of a graph, their 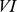 depends only on the disagreement of clusters in that region. It is easy to see that the 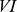 between two clusterings with 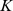 clusters is such that:
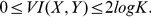
(8)


This implies that the maximum 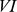 distance grows like 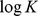. In particular when 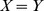, it results 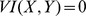. We defer to [16] for further details.

Our simulation scheme consists of two cases: a first scenario (scenario 1) with 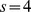 communities of lists, and a second scenario (scenario 2) with 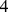 communities and 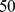 outlier lists for a total of 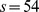 communities. In the following we report the 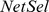 clustering results averaged over 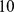 simulation runs for each scenario. We also provide a comparison to a classical clustering algorithm, 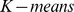, using the number of mismatches as distance. We notice that, when the number of true communities is 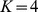, equation (8) implies 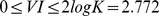.

#### Scenario 1

In the first scenario we populated each of the 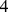 communities of lists, by 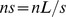 lists, where 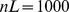 is the total number of lists in each simulation run. In order to explore the sensibility of 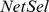 with respect to the parameters 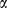 and 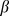, we generated two subcases, namely 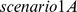 and 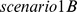.

In 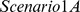 we allowed 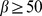 and 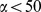. In this case the variation of information between the clustering obtained by 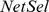 and the true one is always zero (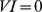). This is due to the capability of 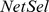 to recover the true community for each of the simulated lists. In particular table 1 and 2 show the values of the indicators 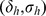 and, respectively, 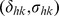 for the 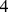 communities of lists simulated with 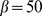 and 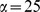. For sake of comparison we applied 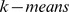 clustering on the same example, and we found that despite it finds the correct number of clusters, the truth is only partially recovered. Indeed the variation of information between k-means clustering on this example and the truth is 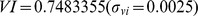.

In 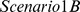 we allow 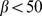 and 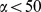. This scenario depicts the situation in which each couple of lists shares at least the 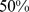 of information. Thus even if we are generating four separate clusters, all the lists actually belong to the same group. In this case 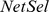 outputs a unique cluster thus always yielding 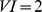, while 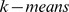 randomly clusters the lists. As an example the average result on 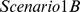 with 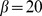 and 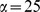 is 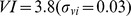. Observe that in this case the 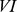 boundary condition (8) does not hold, because the two clustering compared do not have the same number of clusters. The results obtained on the 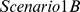 suggest that when the lists are not well separated, in the sense that they share an high amount of information, they should be considered as a unique true cluster. In this case it is correct to directly apply classical techniques for rank aggregation so to merge them together.

#### Scenario 2

In order to highlight the capability of 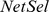 to isolate the outliers, we simulated a second scenario composed of 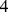 communities and 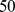 outlier lists, for a total of 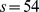 communities. Also in this scenario we generated two subcases, namely 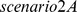 and 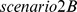.

In 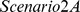 we allowed 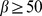 and 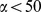. In this scenario 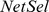 always correctly identified the 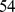 communities. Thus, also in this case, the variation of information between the clustering obtained by 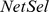 and the true one is always zero (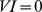). This is due to the capability of 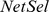 to recover not only the true community for each of the simulated lists, but also to identify each outlier as an isolate community. In particular Tables 3 and 4 show the values of the indicators 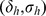 and 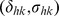 respectively for the 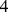 communities of lists, simulated with 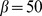 and 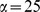, apart from the outliers. The 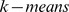 clustering on the same example correctly identifies 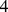 communities but is not able to isolate the outliers, in the sense that they are all assigned to a same true communitiy. The variation of information between k-means clustering on this example and the truth is 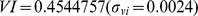.

In 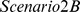 we allowed 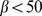 and 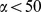. According to the deductions from 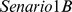, also in this case 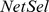 fails to detect the true four communities. Indeed all the 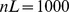 lists belonging to them are associated to a unique cluster. Nevertheless 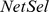 surprisingly identifies each of the 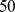 outlier as an isolate community. The variation of information of 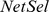 relative to 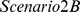 is always 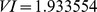. On the contrary the 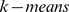 clustering on the same example is completely random. As example the average result on 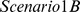 with 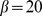 and 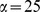 is 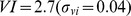.

As main result we get that 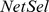 is robust with respect to the variability within the same group. On the other hand it is strongly influenced by the percentage of mismatches between groups. In fact, for any of the tested values of the parameter 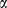, only when 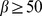, our method perfectly picks the true original communities. The other way round, when 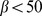, our algorithm fails to detect the underlying simulated community structure as all the lists are assigned to the same community. This is due to the true nature of the simulated data set that is composed of similar lists in terms of mismatches. In this case our method is not well suited and thus we suggest to use a more specific custom strategy. Another strong property of 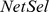, highlighted by the simulation scheme, is the capability of 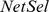 to isolate the outliers in any scenario.

### Robustness

In the subsection 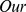 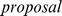 we showed that the third step of 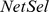 is devoted to the extraction of the communities of similar lists. This step was performed via 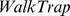, a dynamic algorithm for community detection based on random walks [7]. Since algorithms for community detection are still object of very active research, in this subsection we will show that 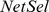 final results do not depend on the community extraction technique applied. To this end we compare the overall performance of 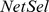 on the 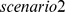 to the one obtained employing other three community detection methods: 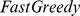 [18], 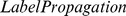 [19] and 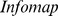 [20]. 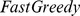 is an algorithm based on the greedy optimization of the quantity known as modularity [21]. 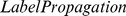 is a simple and fast method based on the iterative propagation of communities labels across the graph. We chose these algorithms because, according to the categorization given by Fortunato in [8], each belongs to a different category of community detection methods. Indeed 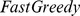 is a modularity based algorithm, 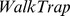 is a dynamic method, while 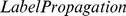 is a sort of standing alone alternative method. Moreover we also explore the performance obtained employing 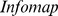, the dynamic algorithm by Rosvall and Bergstrom [20], as community extraction method in the third step of 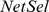. We included 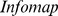 in our comparative analysis because Lancichinetti and Fortunato [22] show that it is very reliable, and they suggest to adopt it as a first approach, especially when no specific information on the network at study is available. Experimental results on simulated data show that the communities detected by 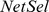 is invariant under the application of either 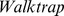, 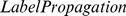, 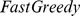 or 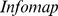. Hence we defer to the previous subsection 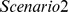 for the description of the results in terms of 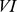 and indicators 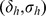 and 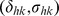. This is a strong indication of the robustness of 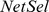 with respect to the community extraction algorithm applied in the third step.

### Application to real data

In this section we report the empirical evidences achieved on two real data examples: a financial dataset and a biological dataset. We have decided to use financial data because, to our knowledge, there are not contributions in this direction in the field of credit risk analysis. Moreover we also show the potentiality of 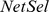 in the bioinformatics field, providing an application to a biological microarray dataset. Indeed microarray data can be interpreted as a set of ordered lists of genes and so analyzed via rank aggregation methods as suggested in [4] and [23].

#### Financial dataset

The real financial data set is composed of 1000 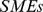 (Small and Medium Enterprises) and a set of financial ratios (lists) expressing a ranking on them. For a clear description of this data set, the reader can refer to [24]. Considering the real data at hand, first we run logistic regression and classification tree on all the lists (financial ratios) available. For sake of comparison, we also build the same two models only on the subgroup of lists selected by 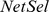. In the following we show that, on the basis of performance indicators on predictive power, the predictive models built on the subgroup of variables selected by 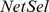 outperforms the same models built on the complete set of financial ratios.

In order to introduce the application on real financial data, we recall that credit is the loan that can only be granted by authorized financial institutions or banks to the customer who applies for credit. After a credit application is taken by a creditor, an assessment process is performed in order to decide whether to approve or reject grating credit to the applicant, depending on the registered customer information expressed by quantitative and qualitative statistical variables. In finance literature, this process is known as credit scoring that is a classification method aiming to distinguish the desired customers who will fully repay from defaulters.

There have been several supervised methods applied to credit scoring of customers in literature such as discriminate analysis, linear regression, logistic regression, non parametric smoothing methods (i.e. Generalized Additive Models), genetic algorithm, neural networks, graphical models and others (see e.g. for a review [25] and [26]).

We underline that supervised classification aims to construct a rule for assigning a score which represents a risk for each statistical unit, on the basis of a set of available lists (financial ratios).

In order to predict the probability of default, 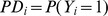 for every observation 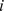 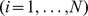, a supervised model for credit risk estimation considers 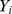 as the objective binary variable and a set of 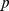 lists 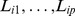. In particular the binary variable 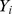 takes value 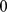 if the customer is good and 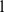 otherwise.

More precisely, a credit scoring model summarizes all the information available measured on the variables in a single list which reports the probability of default for each statistical unit. This means that, starting from a multivariate problem, we derive only one variable which can be used to provide an ordering of risk among the statistical units at hand.

On the basis of our methodological proposal, we think that the results achieved in supervised models can be improved by 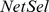 because it takes into account the information on each list, thus providing a better ordering and comparison in the data collected.

We show that using 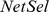, we are able to select groups of lists which provide similar order in terms of risk for the statistical units. This means that our approach leads also to select groups of features highly related to default.

Furthermore, we highlight that 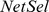 is more robust with respect to data mining with missing data, corrupted data, inconsistent data and outliers.

In our analysis, for every considered statistical unit 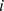 (company), our information consists of a binary response variable 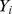 and a set of explanatory variables or lists 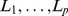. In particular, the data set is composed of companies with negative solvency (default) if 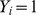 and companies with positive solvency (not default) if 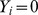.

We have considered the following financial ratios (see e.g. [24]): 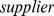 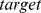, 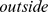 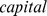 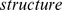, 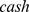 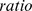, 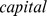 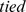 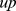, 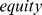 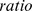, 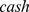 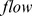 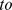 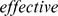 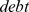, 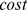 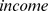 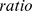, 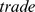 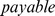 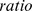, 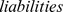 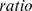, 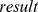 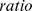 and 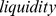 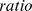.

The prior probability (i.e. number of defaults divided the number of observations) of default is equal at 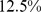. In order to predict the probability of default for each 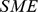, we run both a classical logistic regression model [27] and a classification tree [28] considering the whole set of 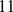 financial ratios.

The logistic regression is a type of regression analysis used for predicting the outcome of a binary target variable as a function of a set of covariates. While logistic regression is a parametric model of the family of the generalized linear models, tree model are non parametric supervised techniques. Since the dependent variable is binary, in this application we have compared logistic regression with classification trees.

The logistic regression selects as significant only two financial ratios, namely 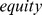 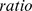 and 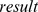 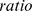. On the other hand, classification tree reports 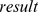 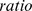, 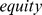 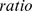, 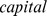 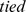 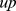, 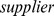 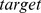 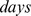 and 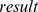 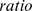 as significant.

In order to select the best model out of these two, we have done a cross validation exercise using 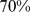 of observations as training data and 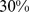 of observations as validation data. We have employed different measures of performances (on the validation set) based on the confusion matrix [28]and assessment indicators as the lift and the response chart (see e.g. [29]).

In order to derive the lift, we put the observations in the validation set into increasing or decreasing order on the basis of their score, which is the probability of the response event (default), as estimated on the basis of the training set. We then subdivided these scores into deciles and calculated the observed probability of default for each of the decile classes in the validation set. A model is good if the observed success probabilities follow the same order as the estimated probabilities.

The other way around, cumulative captured response (CCR) gives the percentage of predicted events for each decile. If the model were perfect, this percentage would be 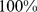 for the first deciles and equal to zero for the other deciles.

The out of sample performance of logistic regression and tree models computed using all the variables available are shown in Table 5. Considering the lift and the cumulative captured response, we choose as best model the classification tree which captures the 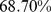 of the event of interest, using only the first three deciles.

We have also considered for each model the 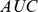 (Area Under the 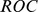 Curve) [30], a classical measure of predictive performance employed to compare logistic regression with classification trees. The receiver operating characteristic (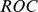), or simply 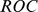 curve, is a graphical plot which illustrates the performance of a binary classifier system as its discrimination threshold is varied. It is created by plotting the fraction of true positives out of the positives (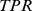  =  true positive rate) vs. the fraction of false positives out of the negatives (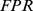  =  false positive rate), at various threshold settings. (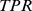 is also known as sensitivity, and 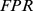 is one minus the specificity or true negative rate). The Area Under Curve (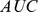) in the machine learning community most often uses the 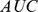 statistic for model comparison. The Area Under the 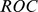 Curve (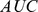) metric has achieved a big success in binary classification problems since they measure the performance of classifiers without making any specific assumptions about the class distribution and misclassification costs.

On the basis of the validation set, we remark that the 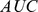 are equal to 0.78 for the logistic regression and 0.85 for the tree model; furthermore, the percentage of correct classifications is equal to 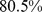 for the logistic regression and 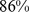 for the classification tree.

However, looking at the nature and the meaning of the financial ratios selected by the logistic regression, we think that 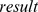 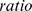 and 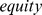 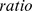 can provide only an idea on how the management is efficient to use its assets to generate earnings and equity. On the other hand, classification tree selects as relevant to predict default a set of features very heterogeneous and different with respect to business practice and expert opinions.

This lead us to investigate a different approach to select the relevant features to do predictive models starting from a set of lists which can generate equal ranking in terms of default forecasting. Moreover the variables selected should have a clear interpretation in terms of business knowledge and expert opinion and should provide also an improvement in terms of predictive performances.

To this purpose we applied 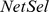 to our set of lists. As shown in Table 6 and in 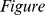 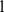, two different groups of variables, 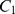 and 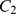, and two outliers, 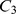 and 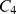, were identified. In particular Tables 7 and 8 show the values of the indicators 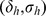 and, respectively, 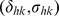 for the communities 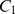 and 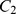.

Expert opinions and business experts confirm that the groups of variables derived using 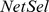 are coherent with business practice (see e.g. [31]) especially for 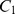 and 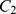.

In order to assess if the groups are also relevant in terms of predictive ability, we have applied logistic regression and classification tree separately on 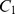 and 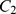. We have tested the models in terms of out of sample performance using the same proportions specified before.

On the basis of the variables in 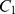, both predictive models perform better with respect to the models build on the whole data set. Table 9 reports the results in terms of lift and cumulated captured response. As we can observe from Table 9 tree model is the best one and, using the first three deciles, it captures the 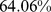 of the events of interest. The AUC values are equals to 0.85 for the logistic regression and 0.89 for the tree model and the percentage of correct classifications is equal to 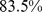 for the logistic regression and 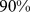 for the classification tree. Finally, we have considered the 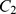 variables to predict default. Both variables are statistically significant for the logistic regression. Furthermore, the logistic regression and the tree models give interesting results in terms of out of sample performance. Table 10 underlines that the tree model is the best one and using the first three deciles it captures the 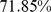 of the events of interest. The AUC values are equals to 0.80 for the logistic regression and 0.87 for the tree model and the percentage of correct classifications is equal to 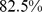 for the logistic regression and 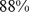 for the classification tree.

Our real application shows that 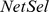 is able to select coherent sub sets of variables highly related to default estimation. As a consequence, the models built on the communities selected perform better in terms of out of sample measures with respect to the results achieved on the whole data set.

#### Biological dataset

Rank aggregation techniques is gaining a growing attention in the bioinformatics applications. During the last decade microarrays have become a standard technology to monitoring the activity of virtually all the genes from a biological sample in a single experiment. They offer a unique perspective for explaining the global genetic picture of a biological sample subject to whatever stressing conditions. Nevertheless, the result of a microarray experiment is often summarized in terms of a ranked list of genes differentially expressed between two conditions. This list of selected genes (usually hundreds) needs then to be explained, but the automated translation of the list into a biological interpretation is often challenging. Given that a microarray experiment can be interpreted as a set of ranked list, it is suitable to be analyzed by 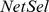. In order to provide an example of such an analysis, we selected the dataset 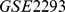 from 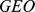 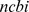 database (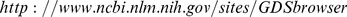). This dataset collects the expression data of a selected suite of 192 metabolic genes measured on three tissue (brain, heart, and liver) from three individuals among three different natural populations of 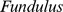 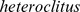 using a highly replicated experimental design, as it is described in [32]. In particular, each 3 individuals were respectively collected from Maine, New Jersey, and Georgia. Each of these 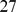 samples was measured four times, twice with 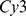 (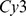 green fluoresce dyes) and twice with 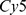 (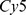 red fluoresce dyes). A total of 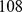 hybridizations were performed (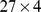), and hence the corresponding 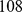 expression values ranked lists would be considered as the nodes of a graph. Given that we know the true labels of each node in terms of population (Main, New Jersey and Georgia) and in terms of tissue (brain, heart and lung), it would be very interesting to know if 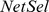 is able to provide insight into the variation in tissue-specific gene expression among individuals and among different natural populations of a species. The partition recovered by 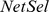 consists of three communities (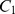, 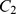 and 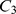) and is summarized in Table 11 and in the pie plot in 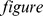 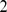. In Table 11 we report the percentage of tissue samples assigned to each community. As you can see the majority of liver samples (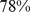) are classified in the same community 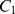, while Heart and Liver samples populate community 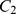 and community 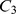 with very close percentages and are almost absent (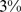) in community 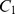. 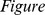 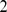 show a pie plot of the same percentages detailed in Table 11. In particular we used green dye for brain, red dye for liver and blue dye for heart. Each color has three intensities in order to label the three population: light for the New Jersey (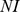), medium for the Maine (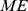) and dark for the Georgia (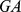). It is evident that liver samples shares the most similar expression values apart from the population and the variation between individuals. At the same time liver expression values depart from brain and heart one. The other way round we observe that brain and heart expression values are almost similar. This lead us to the interesting conclusion that the majority of the genes under study are liver specific and expression profiles are highly varying among individuals and populations. Moreover, this result is confirmed in the original study presented in [32], where it is shown that liver-specific expression accounted for 61% of the expression differences among tissues. Heart-specific and brain-specific expression accounted for 24% and 15% of differences among tissues, respectively. Furthermore they show that, regardless of population, expression patterns were typically most similar between heart and brain, and least similar between liver and heart.

### Stability

In this section we will examine the stability of the partition recovered by 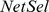 on the two real datasets against random perturbations of the graph structure. To address this issue we specify an intuitive empirical method for perturbing a network by an arbitrary amount. Mimicking the approach proposed by [33], we restrict our perturbed networks to having the same numbers of vertices and edges as the original unperturbed network, hence only the positions of the edges will be perturbed. Moreover, we expect that if a network is perturbed only by a small amount, 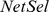 partition will have just a few edges moved in different communities, while a maximally perturbed network will produce completely random clusters. In [33] the perturbation strategy is achieved by removing each edge with a certain probability 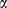 and replacing it with another edge between a pair of vertex 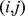 chosen at random with a probability proportional to the degree of 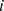 and 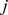. Varying the probability 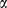 from 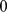 (original graph) to 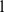 (maximal perturbation), many perturbed graph are generated and compared to the partition on the original graph by means of 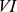. Our simplified version of this perturbation strategy consists in randomly permuting a percentage 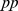 of edge from the original graph obtained at the 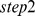 of 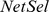. Again a null percentage of permutation 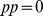 corresponds to the original unperturbed graph, while 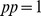 corresponds to the maximal perturbation level and thus we consider the corresponding random graph as the null model. Following [33] we generated many (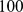) perturbed graph at different levels of 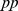 varying from 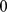 to 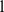. We then computed the 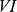 between the cluster structures identified by 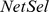 on the perturbed graph to the 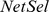 partition obtained on the original graph. 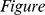 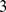 and 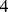 show the average results of the application of our stability analysis to the two real data example discussed previously. The figures depict the average value of the normalized variation of information as a function of the amount of perturbation 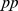. Both the figures show that the normalized variation of information starts at zero when 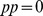, as it corresponds to the 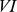 between the unperturbed starting network and itself, grows rapidly and then flats as 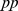 approaches its maximum value of 1. The black points in the figures show the variation of information for the real network while the red points show the results for the correspondent random graph. It is easy to see that in both cases the 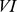 for the real data curves depart significantly from the null model, strongly supporting that the community structure discovered by the algorithm is relatively robust against perturbation. In order to loosely interpret the results, we can assume that the value of the 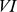 corresponds to the percentage of vertices assigned to different communities between the original and the permuted graph partitions. In this light the two figures includes two horizontal lines referring respectively to 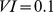 and 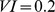. For example, in 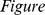 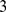 the curve for the real financial network crosses the line representing reassignment of 20% of the vertices close to the point where 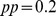 meaning that about 20% of the edges must be permuted before 20% of the vertices are assigned by 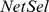 to different communities. On the other hand, only about 5% of the edges of the random graph need to be permuted to reach this point.

## Discussion

In this paper we propose 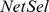, a novel methodology for discovering homogeneous groups of rankings. We describe our proposal in a theoretical framework and we also provide an effective algorithm. The implementation of 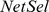 is written in the statistical programming language 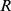 and is available on demand. On the basis of an extensive simulation activity, we prove that, when dealing with a non homogeneous set of lists, our approach outperforms related methods proposed in the literature. Finally, testing on real financial data shows that 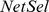 is a powerful approach able to improve predictive performances in credit risk analysis. Moreover, the application of 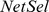 on a real biological dataset gives an idea about the contribution that our method could provide in the bioinformatics field.

Our method is easy to implement, does not have computational overhead and is able to isolate outliers. However our methodology reveals uninformative in case of a unique group of homogeneous set of lists. Indeed 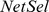 is able to detect a connection between two lists only if the degree of similarity between them is almost a least the 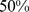. As a consequence, 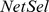 is not sensible to moderate differences between lists and would produce a unique cluster in such cases. Another important aspect to emphasize and discuss is that 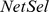 is designed only for graphs whose nodes are ranked lists, thus we could not test it on artificial networks like Girvan-Newman [34] and 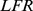 [35], [36] benchmarks. Furthermore, to the best of our knowledge, real data networks of ranked lists already analyzed in the literature are still few and they have been analyzed by numerical and statistical methods not designed for the community extraction. This implies that a direct application of 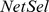 on real networks needs to be discussed and validated each time without the comparison with literature methods. Future work would focus on measuring the efficacy of 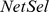 as a variable selection method when the variables can be interpreted as orderings.
